# Phenotypic, genetic, and genome-wide structure in the metabolic syndrome

**DOI:** 10.1186/1471-2156-4-S1-S95

**Published:** 2003-12-31

**Authors:** Lisa J Martin, Kari E North, Tom Dyer, John Blangero, Anthony G Comuzzie, Jeff Williams

**Affiliations:** 1 Center for Epidemiology and Biostatistics, Cincinnati Children's Hospital Medical Center, Cincinnati, Ohio, 45229 USA; 2 Department of Epidemiology, University of North Carolina, Chapel Hill North, Carolina, 27514 USA; 3 Department of Genetics, Southwest Foundation for Biomedical Research, San Antonio, Texas, 78245 USA

## Abstract

**Background:**

Insulin resistance, obesity, dyslipidemia, and high blood pressure characterize the metabolic syndrome. In an effort to explore the utility of different multivariate methods of data reduction to better understand the genetic influences on the aggregation of metabolic syndrome phenotypes, we calculated phenotypic, genetic, and genome-wide LOD score correlation matrices using five traits (total cholesterol, high density lipoprotein cholesterol, triglycerides, systolic blood pressure, and body mass index) from the Framingham Heart Study data set prepared for the Genetic Analysis Workshop 13, clinic visits 10 and 1 for the original and offspring cohorts, respectively. We next applied factor analysis to summarize the relationship between these phenotypes.

**Results:**

Factors generated from the genetic correlation matrix explained the most variation. Factors extracted using the other matrices followed a different pattern and suggest distinct effects.

**Conclusions:**

Given these results, different methods of multivariate data reduction may provide unique clues on the clustering of this complex syndrome.

## Background

The metabolic syndrome (MS) is a cluster of abnormalities including central obesity, abnormal glucose tolerance, elevated insulin and triglycerides, and depressed HDL-C [1-3]. Previous epidemiological studies have implicated common underlying factors influencing the clustering of this syndrome [4]. Yet, the metabolic, physiological, and genetic mechanisms responsible for this clustering have not been elucidated.

Because major genes involved in the etiology of common complex diseases are likely to exert an effect on multiple quantitative traits, statistical techniques that permit the joint analysis of correlated traits, such as factor analysis, may aid in analysis [5]. Using factor analysis, heritable clusters of MS traits have been identified based on phenotypic relationships [6,7]. To our knowledge, no studies have used the genetic correlation matrix to construct factors for MS traits. Related to this, no studies have explored the use of a 'genome-wide' correlation matrix as an alternative to the phenotypic and genetic correlation matrices. Direct manipulation of the genetic and genomic correlation matrices could represent a powerful method for elucidating the genetic architecture of multiple complex traits. In this study, therefore, we investigated genetic influences on the aggregation of MS phenotypes by applying a uniform factor analytical method to phenotypic, genetic, and genome-wide ('genomic') LOD score correlation matrices using five phenotypic traits (total cholesterol (CHOL), high density lipoprotein cholesterol (HDL-C), triglycerides (TG), systolic blood pressure (SBP), and body mass index (BMI)) from the Framingham data set prepared for the Genetic Analysis Workshop 13 (GAW13).

## Methods

### Data

The Framingham Heart Study was initiated in 1948 and consisted of 5209 men and women between the ages of 30 and 62 recruited from Framingham, Massachusetts. The subjects returned every 2 years for a detailed medical history, physical examination, and laboratory tests. In 1971, a second-generation group consisting of 5124 of the original participants' adult children and their spouses was enrolled. Longitudinal data were available on SBP, height, weight, CHOL, HDL-C, TG, glucose, hypertensive treatment, hypertensive status, number of cigarettes smoked per day, and grams of alcohol per day. Although glucose was available, we were unable to control for diabetes status, and in the absence of this information the trait was not heritable (data not shown).

The following five phenotypes from the Framingham Heart Study were used to define MS: CHOL, HDL-C, TG, SBP, and BMI. We chose to focus on a single time point for all phenotypic variables. In the original cohort, we used clinic visit 10 because this is the first visit for which data on CHOL and HDL-C were collected. In the offspring cohort, we used clinic visit 1, at which all of the phenotypic data were available and had been collected during a similar timeframe. We also reasoned that by selecting these visits (as early as possible with the data of interest), we could maximize the number of participants included in our analyses. Outliers more than four standard deviations from the mean were dropped; only individuals having complete covariate data (age, sex, cohort, hypertensive treatment, hypertensive status, and smoking) were kept (*n *= 1648).

### Genome-wide LOD correlations

Using the 330 extended families, heritabilities were estimated after adjustment for the above covariates. A variance component model implemented in the program package SOLAR [8], was used to generate multipoint identity-by-descent (IBD) matrices and genome-wide LOD scores. A LOD-score evaluation was performed every 10 centimorgans. Using SAS [9], we calculated a correlation matrix from the genome wide LOD scores.

### Phenotypic and genetic correlation matrices

We used bivariate variance-component analysis to estimate the phenotypic, genetic, and environmental correlations between all pair-wise combinations of traits. This method has been described in detail elsewhere [10,11]; but briefly, the phenotypic covariance is modeled so that the covariation between two individuals for two traits is given by a 2 × 2 covariance matrix with the elements defined by:

Ω_*ab *_= 2Φρ_*G*_σ_*ga*_σ_*gb *_+ Iρ_*E*_σ_*ea*_σ_*eb*_, (1)

where *a *and *b *take the values of 1 or 2 and ρ_G _and ρ_*E *_are the additive genetic and environmental correlations between the traits. The genetic correlation estimates the proportion of genes shared in common between the traits. This approach has been implemented in SOLAR version 2.0. The phenotypic correlation (ρ_P_) is given by:



where  and  are the heritabilities of the traits. These correlations were assembled into phenotypic and genotypic matrices for factor analysis.

### Factor analysis

The genetic, phenotypic, and genomic correlation matrices were factor analyzed to summarize the relationships between the five phenotypes in the MS using SAS [10]. Orthogonal factors that are linear combinations of the original phenotypes are constructed that explain as much of the total variance in the original variables as possible. Factors were varimax rotated, and factor loadings of 0.40 or greater were used to interpret the factor structures [12,13].

## Results

**Figure 1:**
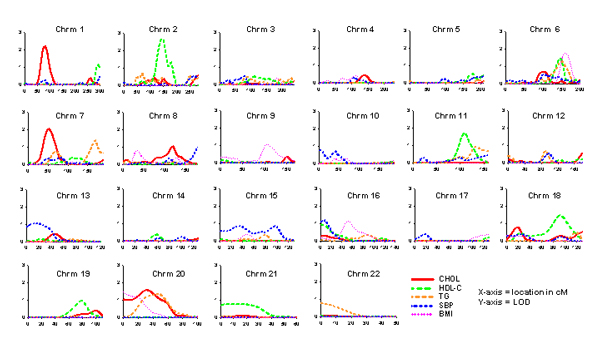
Genome-wide LOD scores for CHOL (red), HDL-C (green), SBP (blue), TG (orange), and BMI (pink).

Heritabilities were determined to be significant for BMI (38.7 ± 3.9), CHOL (41.5 ± 5.6), HDL-C (41.5 ± 5.6), TG (45.6 ± 5.7), and SBP (16.4 ± 3.5). The LOD scores for the genome scans of the traits are shown in Figure 1. Although there are several suggestive linkages, no LOD scores reach significance [14].

Tables 1 and 2 report the genetic, environmental, genomic, and phenotypic correlation matrices. The rotated factor loadings generated from the genetic, phenotypic, and genome-wide LOD score correlation matrices are summarized in Table 3. Factor loadings greater that or equal to 0.40 are indicated in bold type. The genetic correlation factors explained the most variation with factors G1 and G2 explaining, respectively, 31.3% and 24.7% of the total genetic variance. The pattern of loadings differs across the matrices examined, but in general the first factor in each group appears to be a magnitude axis (all loading in the same direction) with high loadings in each of the categories (lipids, fatness, and SBP). For the genetic and phenotypic factors, HDL-C loads in the opposite direction from the other variables, but given that decreased levels are a risk factor, this is to be expected. For the genomic factor, HDL-C loads in the same direction as the other variables because the genomic correlation is concerned only with whether genomic regions account for variability but not with direction of change.

## Discussion

Previously, factor analysis has been used to identify components underlying the MS through the construction of factors from phenotypic values. Because factor loadings from the genetic and phenotypic correlation matrices are distinct, however, reliance on phenotypic correlation alone may fail to disclose underlying genetic relationships.

In this study we constructed factors not only from phenotypic correlations, but also from the genetic and genome-wide LOD score correlations. Factors extracted from these correlations exhibited variable structure and suggest distinctive effects. With the exception of the second factor from the genome-wide LOD score correlation matrix, SBP loaded strongly on every factor. In other studies, however, SBP has not loaded strongly with other components of MS [6,7]. Because we were unable to consider glucose or insulin, and because the properties of the variables chosen for analysis can unduly influence the results [15], it is not known whether SBP would remain as pivotal when considered in combination with glucose or insulin.

However, as the genetic correlations are estimated from a polygenic model with no major gene effects estimated, it is possible that the first factor from the genetic correlation matrix is simply summarizing the polygenic effects between the traits. Similarly, the second factor may summarize the QTL effects; indeed, the second factor of the genetic correlation matrix loads similarly to the genome-wide LOD score correlation matrix that summarizes the correlation of QTLs across the genome.

## Conclusions

In summary, factors extracted using the phenotypic, genetic, and genome wide LOD score correlation matrices followed different patterns and may suggest distinct effects. Thus, these results imply that different methods of multivariate data reduction provide unique clues on the clustering of this complex syndrome.

## Authors' contributions

LM and KN performed statistical analyses and interpreted results. JW assisted in the interpretation of the results. TD calculated the IBDs. JB and AC participated in the design of the study. All authors read and approved the final manuscript.

## Figures and Tables

**Table 1:** Genetic (above diagonal) and environmental (below diagonal) correlation matrices ± standard error.

	**CHOL**	**HDL-C**	**SBP**	**TB**	**BMI**
CHOL		-0.06 ± 0.09	0.04 ± 0.11	0.32 ± 0.08	0.11 ± 0.08
HDL-C	0.27 ± 0.06		-0.22 ± 0.12	-0.46 ± 0.09	-0.13 ± 0.09
SBP	0.02 ± 0.05	0.13 ± 0.05		0.29 ± 0.11	0.01 ± 0.11
TG	0.38 ± 0.05	-0.24 ± 0.06	0.02 ± 0.05		0.03 ± 0.09
BMI	0.06 ± 0.05	-0.24 ± 0.05	0.22 ± 0.04	0.29 ± 0.05	

**Table 2:** Genomic (above diagonal) and phenotypic (below diagonal) correlation matrices.

	**CHOL**	**HDL-C**	**SBP**	**TB**	**BMI**
CHOL		0.01 ± 0.05	0.06 ± 0.05	0.25 ± 0.05	0.14 ± 0.05
HDL-C	0.12		0.08 ± 0.05	0.19 ± 0.05	0.01 ± 0.05
SBP	0.03	0.03		0.07 ± 0.05	-0.03 ± 0.05
TG	0.35	-0.34	0.10		0.23 ± 0.05
BMI	0.08	-0.20	0.16	0.18	

**Table 3:** Factor loadings from the genetic, phenotypic, and genomic LOD score correlation matrices.

	**Genetic**		**Phenotypic**		**Genome-wide**	
			
	Factor 1	Factor 2	Factor 1	Factor 2	Factor 1	Factor 2
CHOL	0.30	**0.66**	0.17	**0.91**	**0.65**	0.04
HDL-C	**-0.77**	0.10	**0.91**	0.06	0.08	**0.73**
SBP	**0.76**	**0.41**	**-0.52**	**0.68**	**0.71**	0.32
TG	**0.57**	-0.01	0.15	0.01	-0.04	**0.68**
BMI	0.39	**-0.67**	**-0.41**	0.09	**0.69**	-0.22
% Var	31.3	24.7	27.9	22.5	25.1	18.9
